# Reviews and Book Notices

**Published:** 1877-12

**Authors:** 


### Reviews and Book Notices.

Transactions of the Twenty-seventh Anniversary Meeting of the Illinois State Medical Society, Held in the City of Chicago, May 15, 16, and 17, 1877. Chicago: *A. S. Kissell & Co.*

At the last meeting of The Association of American Medical Editors, Dr. H. C. Wood, in his presidential address, threw out the suggestion that medical societies should abandon the publication of their transactions in volumes. "In these books," he said, "a few kernels of wheat are so buried under an enormous heap of chaff, that they are often entirely lost to the profession. The societies should select some medical periodical, and publish in it the essential part of the proceedings and the meritorious papers. Thus incorporated in a medical journal, the transactions would reach a larger circle of readers, and present in a concise form more wheat and less chaff."

He who has read the last transactions of the Ill. State Med. Society, will heartily wish that Dr. Wood's proposition had been adopted by it; for he finds that he has waded through 250 pages, when a pamphlet, one-fourth smaller, could contain all that is worth printing.

The bulk of the transactions is made up of the reports of the standing committees. The by-laws charge these committees with the duty of preparing an annual report on the important improvements and discoveries in their respective departments. These reports should be brief and concise, in order to be intelligible and interesting; but, in the first place, they should be written by men who command the literary means to collect the necessary material, and possess the time and mental capacity requisite to reduce it to proper form in writing.

If, in the selection of the committees, these conditions are lost sight of, the result is most disastrous. The report is either an *abortion*, such as the present *Report on Practical Medicine*, or a *monster*, such as the *Report on Drugs and Medicines.*

The latter is the most insignificant of all the results which are supposed to be aimed at in a committee report. It comprises sixteen pages in all, and nine of these are filled with phraseology, the profound wisdom of which will best be displayed by a few quotations. On page 105 the reporter startles the reader with the announcement that "the discovery of oxygen by Priestly, in 1772, was a grand era in chemistry;" and on page 106, he continues with this metaphorical effusion: "This element (the oxygen) is the key in the arch of nature's combinations, and its discovery led to the analysis of many mysterious conditions, as it seemed the combining link, the lever which opened the seal of secrecy, the cypher by which many things unexplained could be wrought out and made plain. It was the opening wedge to future investigation, as the key of explanation had been found." What a nondescript we have presented to us in that which is at once a *link*, a *wedge*, a *lever* and a *key!*

This compound of nonsense and bombast is followed by three pages of abstracts from the *Journal of Davis and Leonard, an advertising sheet, published for the benefit of Parke, Davis & Co., of Detroit!* To appropriately crown his wonderful work, the writer concludes with a terrible tirade against the introduction into pharmacy of the metrical weights. He objects on account of the —— orthography! Certainly *he* does not seem to be on very good terms with it. One might even conclude that the doctor confesses that tailors and grocers know more about it than he himself; for on page 118 he says, "This metric system will be appropriate for tailors and machinists, where length and breadth are to be estimated; or for lawyers in charging for briefs (*sic!*) it would be excellent for *grocers*, *green*-grocers, to weigh by, but not for druggists or physicians, where life is the issue!"

4

To use the doctor's own language (p. 117), "it is impossible to register all this rubbish;" but the signature he writes under one prescription is too good to be lost to posterity. Perhaps it is "the *link*, the *wedge*, the *key*, the *lever*" to elevate us to the mystical heights of the writer's ideas on the metrical system. It reads thus (page 117): "M. Sigda 1 metri spoonful. 50 deci-hora."

And such doubled-distilled absurdities are permitted to be read, yea to be published, as a report of a standing committee of a State Medical Society!

It is a great relief, indeed to find in the *Report on Surgery*, common sense, plain, unvarnished language and intelligible criticism. The paper, however, though good, is not a committee report. The author could, just as well, have presented it as a volunteer essay. It records a number of interesting cases, each of which involves a question of highly practical importance. A successful reduction of a femur which had been dislocated eleven weeks, gives the doctor an occasion to encourage the attempt at reduction in old dislocations. "I am of the opinion, it is our duty to undertake the reduction of dislocations generally, after a longer period has elapsed than what is laid down by authors as justifiable. With the exercise of ordinary judgment and common sense, no harm can follow from the attempt, and much good may result."

In dressing an injured elbow joint, when impairment of motion is liable to result, the reporter advises not to adhere to any fixed rule as to the position of the limb; but to bandage the arm in that position (either flexed or straight) in which it will, if anchylosed, be of the best use considering the patient's avocation.

Esmarch's bandage, the reporter thinks, has been generally adopted, perhaps "without duly considering the possible hazards attending upon it." He fears that death of the tissues must be the result of even a short interruption of the circulation; and in corroboration of his view, he reports a case of "gangrene and death resulting from the use of Esmarch's bandage." The patient, a young man, had a true anchylosis of the knee joint with the leg flexed well back on the thigh. "The patient was put under the influence of chloroform, and I applied Esmarch's bandage, and, in order to the better secure the femoral artery, I carried the bandage up to Scarpa's triangle, and applied the rubber cord at the proper place for compressing the femoral artery with a tourniquet." A V-shaped piece of bone was removed, of sufficient size to allow the straightening of the leg. The bones were wired on each side; the flesh-wound, except at the angles, closed by silver sutures, and the limb laid on a Liston splint. On the third day after this operation the record reads: "Still doing well, pulse 89. Up to this time patient had not complained of pain, had no fever, and everything so far as I could discover, indicated a speedy recovery." Next morning the patient became delirious; his foot was found cold and black; and the leg, to within four inches of the wound, was discolored and blistered. Amputation above the knee revealed the fact that "all the adipose tissue, from the skin to the muscle, was gangrenous, and a greenish fluid was escaping from its surface." A re-amputation of the thigh at the upper third, gave a healthy stump; but three days later the patient was dead. The reporter's theory of the cause of death is this: Esmarch's bandage caused a paralysis of the limb, and disturbed the nutrition of the tissues so thoroughly that gangrene ensued.

We fail to note the force of this logic, and are rather inclined to see in this case the result of an undetected phlegmonous inflammation with ultimate septicæmia. If the use of Esmarch's bandage had made such profound impression on the innervation and nutrition of the limb as to result in gangrene, then we are at a loss to account for the apparent well-doing of the limb during the first three days; nor can we understand how "*the wound had all healed by first intention*" (as the examination of the amputated limb showed), while mortification was going on in the same tissues. To draw any positive conclusions from a case reported in such a manner as this, seems hardly warrantable in view of the fact that Esmarch himself, as well as others, fully appreciating the danger of suspended circulation have, by a series of careful experiments, determined the length of time during which the limb may be deprived of its nutritive fluid with impunity.

The *report on Idiocy* has one great merit—it is very brief.

The *report on Electro-Therapeutics* can be commended to the chairmen of other committees, as a sample of what a summary report should be. It is clear, complete, and interesting.

The *report on Diseases of Children* has, by some mysterious transformation, been changed to a statistical report of the last scarlatina epidemic in Chicago. We are informed that this epidemic was not so terrible as one would naturally have supposed, after all the hue and cry that it created in this community, about one year ago.

The *report on Otology* consists of remarks on aural inflammations, and contains nothing but what can be found in a better and more complete form, in the standard works on Diseases of the Ear.

When the reports of committees are to be allowed to degenerate in so pitiful a manner as that exhibited in the several reports of the volume before us—it is time for a society to think of remodeling the working plan of its meetings. And the committee which was appointed for that purpose by the last convention, should earnestly weigh the question whether the Illinois State Medical Society would not fare better by abolishing the committee reports and depending solely on volunteer papers for scientific pabulum. We do not admire the system of constrained labor anywhere, and we further believe that when applied to literary efforts, it becomes a nuisance. Put a physician who has never had any ambition for writing essays, at the head of a standing committee, and he feels himself constrained to show "his appreciation of the honor conferred upon him;" he will go into labor and deliver himself of a few pages of grammatical blunders and general absurdities, dished up under the guise of a committee report.

Let those men come to the front who really have something to say; who desire to communicate some new observations, and to impart the result of original researches to their colleagues.

The transactions contain several very creditable essays of this kind; e. g., the paper on "An Improved Method of Performing the Radical Operation in Empyema," by E. F. Ingals, (published in the June number of the JOURNAL AND EXAMINER); the observations on "Apocynum Cannabinum," by Dr. Ransom; a very interesting review of the latest researches on the "Trophic Action of the Nervous System," by Dr. J. S. Jewell; the observations on phimosis as the cause of grave neuroses in children, by J. P. Matthews; the description of a simple apparatus for the treatment by extension of chronic inflammation of the ankle joint, by Dr. E. Andrews. These are contributions in the right direction. They exhibit original ideas, present novel features, and open a field for scientific discussion.

Printed in the same volume with such essays, how insignificant and needless do some other volunteer papers appear. We are really tempted to ask who is to be benefited by such papers as that on "Inflammation of the Middle Ear," which does not nearly compare with the corresponding chapter in Roosa's text-book; or that on "Canthotomy and Some of its Advantages," a paper which contains nothing new. The paper on "Iritis and Some of its Dangers," also disappointed us. We expected to learn here of some hitherto unobserved dangers, but found the whole substance of the paper dwindle down to the observation that an iritis, if neglected or improperly treated, is apt to impair or destroy the sight by occlusion of the pupil. Why must this be told to the members of a medical society in 1877, when it has been an undisputed fact ever since a knowledge was gained of the diseases of the eye?

A prominent feature of the transactions of the Ill. State Med. Society has always been the abundance of typographical errors. And the committee on publication has done its best to preserve in the present volume, the characteristic feature of its predecessors. Besides scores of minor errors (such as *diptheria*, *fœted*, *sulphites* and *ehildren*) we notice a number of others which reflect severely either on the care or on the orthography of the proof-readers. From a large collection we select the following specimens: Page 17, *empyemia* and *empyœmia*; on the same page *Richards* for Richey; p. 58, *Accasia;* p. 60, ferri *murius;* p. 76, *femural,* and *Sharpie's* triangle for Scarpa's; p. 82, *intussesption;* p. 107, *Eccrasceur;* p. 112, *habit* for habitat; p. 115, vis medicatrix *natura;* p. 128, ceteris *parabus.* The headings of pages 175, 177 and 179 bear the legend, "HYGIENICS OF CHILDREN ON PHIMOSIS"! to say nothing of the hieroglyphics intended to represent the formulæ for prescriptions. In every one of these prescriptions some drugs are named in English and others in Latin terms, with a bold defiance of grammar that is truly sublime.[1]

The carelessness in proof-reading is only surpassed by the want of good judgment displayed by the committee on publication, in printing papers which should never have been given to the medical public, or at least not in their present form.

Of course all the papers which were read before the convention, and many which were *not* read, were referred to this committee, whose special duty it is to put all such material in proper shape for publication, after excluding that which is manifestly worthless.

But what shall we think of a committee which publishes fourteen pages of private letters written in answer to the enquiries of the chairman of the committee on practical medicine; letters which show upon their face that they were not intended for reading in public, and which, fortunately for the convention, were not read. They were all referred to the publishing committee, with the request to publish as much of them as was thought proper or necessary. *Not an iota has been changed in these letters,* nor in any other paper presented, although the crude form of many of these compositions would have been greatly benefited by the editorial file.

We have penned this criticism prompted solely by a feeling of profound regret. The Illinois State Medical Society is composed of the great body of professional men in our own State. We have, so to speak, a personal interest in this home institution, and anxiously desire that its Transactions should be such as we can regard with pleasure, consult with profit, and issue to

---

1 E. g. Page 52, Aqua Camp. ℥iv; p. 62, Mucilago Amyle ℥ii!

the world with pardonable pride. For this reason we have tried to show in the foregoing pages, how far below this standard the present volume of Transactions has fallen. It is better, far better, for us to learn of its fatal defects and deficiencies at home, than to be informed of them in the cruel phraseology of some strange critic, who may think the volume really worth dissecting. F. C. H.

LECTURES ON PRACTICAL SURGERY. By *H. H. Toland, M. D.*, Professor of the Principles and Practice of Surgery and Clinical Surgery, in the Medical Department of the University of California.

The Pacific coast is the home of the bonanza. As a work on surgery, of any kind, this book is a bonanza of curiosities in style, argument and statements.

The very essence of practicality is in being systematic and definite. This book possesses no system whatever, either in its treatment of general subjects or in specification. It is not definite or explicit in anything except dogmatical assertions and examples of the personal infallibility of the author.

It opens with a consideration of Anæsthetics and Inflammation. *Ether* is "good for old or very young people." Chloroform should be given "rapidly," in order to avoid its after effects upon the blood! We had supposed the general impression to be, that chloroform within limits was less harmful in proportion to the youth of the patient.

The pathology of inflammation, as given, is notably weak, loose and behind the times. Three stages of termination are laid down: "1st. Dilitescence! 2d. Resolution. 3d. Mortification! In the first the inflammation "absconds," like a thief at night, and the "parts become natural." In the second the parts become natural honestly, as it were,—we fail to appreciate the benefit or the importance of the distinction.

Following mortification, "no amputation should ever be performed until the line of demarkation has fully and deeply formed," so that "a division of the bones" only will be required. The first amputation we ever witnessed was for acute spreading gangrene, and the patient's life was undoubtedly saved by the operation. It is certainly a well recognized and proper procedure in some cases of this nature. Hospital gangrene was never heard of on the Pacific coast.

The remarks on the general treatment of inflammation certainly impress one with the idea that the powers of nature and the grace of God have no business to interfere for the welfare of the patient; if he does not get well per prescription and treatment of the doctor, he does right to "return to his fathers." The treatment advised is emphatically and positively *antiphlogistic.* 1st. General blood letting "*to syncope*;" 2d. Leeches by the dozen; 3d. Blisters large and full; 4th. Calomel *sure*—are indispensable and wonder-working. (Oh! ye shades of departed Esculapians, how ye must rejoice!)

If these *remedies* (?) will not work, or you "be timid," the author furnishes some "especially good" prescriptions, called "depressants" and "deobstruents," (*sic*) and the reader is at the same time notified that they are especially "superior" for "pneumonia and pericarditis;" and *note well*, if the latter trouble "be not *properly* treated it may terminate in death!"

Still a few pages further on, the author again helps us out with another "invaluable" prescription, which will "fulfill the indications more perfectly than any other remedy," in chronic inflammation of "*any* of the abdominal organs"! This last combination—p. 59—looks very much as if it might be the formula from which the universally applicable "California Vinegar Bitters" is concocted. (Quien Sabe ?) This, of course, is "Practical Surgery"! Again, on p. 67, the cure of dropsy and all serous effusions is made easy and certain by a never failing combination, beyond which it is useless to search, or words to that effect.

We believe these things to be entirely out of place in *any* surgery, and when found in a course of "Practical Lectures" to a class of students, they indicate either a want of confidence in the Professor of Practice, or an inane desire on the part of the lecturer to deliver himself of some "sure cures."

On page 74 is the astounding assertion that an "Abscess is formed by the *absorption* of the surrounding parts for its accommodation!" We had always supposed that the "surrounding parts" were displaced merely by the accumulating fluid. If the latter were not the true state of affairs, abscesses would be deadly and dire afflictions, and not commonplace, as they usually are. Again, page 77, "Pus is a secretion," "prepared by the capillary vessels!" There *is* a fountain sure. Again, "Abscesses are frequently absorbed," even "syphilitic abscesses are absorbed" without "any especial contamination of the system"!

Chapter VI. is a useless conglomerate mixture of unsupportable assertions, terrible platitudes, and "rare exceptional cases," under the head of "Lymphization." It is wonderful how the author manages to avoid the subject in hand, and wanders, and wanders on a "still hunt" after new topics, not only in this chapter, but in every chapter. No subject is treated of continuously—nothing exhaustively. He is continually "breaking out" with something else, like the coming forth of new crops of boils—it is really painful.

Chapter VII. opens with a "rare" case of ordinary scrotal hematocele, illustrative of "hemorrhage from inflammation"! Following this we are treated to some of the most unique opinions upon absorption and ulceration that book ever contained. We believe them to be original.

The "same old story" is told in the classification and treatment of ulcers—"Cancer is an ulcer that never heals"! "Ramollissement" uses up one paragraph, which is a whole mint of information on that subject. But does it belong to surgery? On page 93 the author labors with induration, and delivers himself of another formula for enlargement of the spleen and intermittent fever, never known to fail! The remarks on "Transformation," (a new term) "Hypertrophy" and "Atrophy," all in this same chapter, are truly ponderous in their wealth of research and importance of application to the subject in hand, provided the reader allows his imagination to have full play.

Chapter VIII. treats of "Congenital Malformations." Among the first mentioned is "Enlargement of the Fontanelles," for which the liver is blamed, and "three or four grain doses of calomel at night," recommended! The club-foot shoe shown in the plate is the old-fashioned leg brace shoe. A shoe without braces passing beyond the knee joint, and embracing the thigh, can never succeed in correcting the inward twist of the foot in this deformity.

The author's "Felt" apparatus is practically a "fixed dressing," akin to plaster of Paris.

Tumors evidently present a rough and tumultuous sea for the lecturer,—things don't ride easy at all—it can safely be called "Heterologous."

In chapter X. scrofula is duly noticed. The general directions are about up to the accepted beliefs, but the medicine advised consists of the "Vinegar Bitters" prescription, heretofore noticed—it "starts the liver!"

"Never open the larynx or trachea for diphtheria"! This sentence in the author's opinion finally disposes of the important subject of Tracheotomy in this disease—a subject demanding the most careful and painstaking consideration of the practitioner. The opinion is based upon four fatal cases, performed "to gratify the friends" of the patients. This dogmatical statement is alone sufficient to condemn the work; there are many others even worse. In the face of the pathology and treatment of diphtheria, as elucidated and developed by many noted and eminent men—knowing that hundreds of children and adults are now living to testify to the safety and necessity of this operation—we deem this wholesale, sweeping and unqualified assertion—reiterated and repeated as it is in these pages—both outrageous and pernicious. It is valuable only as a monument of the author's total want of information on this subject. No surgeon who waits for the "friends of the patient" to *ask* for the operation before realizing the necessity for it, will ever have any successful cases. The author has a prescription which "dissolves the *albumen*, of which the membrane is composed"!

The consideration of the hip joint, whether with reference to its diseases or dislocations, is lamentable, weak and worthless. Dr. Toland claims to be the introducer of *sub-periosteal*

operations, and yet considers "amputation (at the hip) preferable to re-section," the latter always leaving the limb a "useless, inert mass!"

Under fractures, we find this sentence, "The femur, notwithstanding its *great length*, is *occasionally* fractured"! The author has no use for, and cannot conceive of, any better apparatus for the treatment of fractures of this bone, than the double inclined plane.

Professor Brainard's method of treating ununited fractures is given in this language: Prof. B. "passed ivory pins between the ends of the bones and allowed them to remain," etc.! This *will* be news to Professor B.'s friends.

Tetanus has no terrors for Dr. Toland. He controls the spasms by giving "twenty drops of tr. cannabis indica every two hours," until the "symptoms entirely disappeared," together with morphia.

The author claims the performance of the "first operation for strabismus in America;" the discovery of the hæmostatic properties of Monsel's salt; and to be the first surgeon to perform "sub-periosteal operations." He has also discovered and named a new kind of hernia,—"peritoneal," he calls it. It is apt to affect the position of the linea alba. Chelius' Surgery, published some fifty years ago, contains an excellent description of this same trouble; and the latter surgeon warns the reader not to be led into the mistake that these little protrusions have anything to do with the trouble, if they are found in a patient suffering from colic. Dr. Toland cuts them off, without doing the patient any damage.

The author goes "out of his way" to prove that ophthalmology and otology have shown no advancement during the past "twenty years;" on the contrary, less is known about eye and ear troubles now than then. Such instructions as "bleed from the arm to syncope," "to repeat the bleeding," "apply leeches back of the ears," "shave the scalp and blister," are given as some of the methods of treating "acute inflammation of the eye." Von Graefe and others have done nothing to improve operations for cataract, etc. "Couching" is the best operation.

Under the head of diseases of the joints, a prescription is given for inflammatory rheumatism, which is "the best combination that has ever been suggested," "Dr. Flint, Jr." to the contrary notwithstanding.

The author claims to have performed ligation "for aneurism oftener than any other surgeon in the United States." Somewhere in the book, "the latter end of the last century," is ligated.

Dr. Toland leads many a "forlorn hope" against the opinions of "*other physicians*," whom he has met at the patient's bedside, during his chequered career, and always comes off victorious. This may be an evidence of "Toland's luck," as it is termed; still we cannot think any such reference either modest or properly placed, when found in any scientific work offered to the general profession.

The profession will gain nothing from this work, and like Rip Van Winkle of old, would have been "better off midout it." It is centuries behind the times.

The author, in his preface, calls the work a "Talk" on surgery—yes, an office talk.

We are fully convinced that no surgeon can ever *add* to his laurels by publishing an "*extemporaneous*" book. The last page contains a plate with description of two "rectum specula."

The publishers deserve every praise for the execution of their work. C. T. P.

Observations on the Diseases of the Rectum. By *T. B. Curling*, *F. R. S.*, Consulting Surgeon to the London Hospital. Fourth edition. *Lindsay & Blakiston.* 1876.

We are told in the preface to this new edition, that the former one has been completely revised and added to; so that the work is brought up to date in all matters of which it treats. It is certainly a very excellent work upon a very important subject. It comes from the hands of a surgeon of acknowledged ability and large experience in the department of which he writes; and any one who wants a clever and judicious instructor, and from whom he would seek assistance in the care of cases of diseases of the rectum, cannot do better than to carefully study Mr. Curling's book, and follow its teachings. It is very pleasantly written and beautifully printed and bound; so that altogether the book is a credit to its author as also to the well known publishers.

R. G. B.

Medical and Surgical Reports of the Boston City Hospital. Second series. Edited by *David W. Cheever, M. D.*, and *F. W. Draper, M. D.*, 1877.

This would more correctly be called a second volume, than a second series. Series means a row, and as the first series contained only one volume, though that was a large and valuable one; it was hardly enough to make a row. It is to be hoped that American hospitals will not follow the faulty plan of Guy's, in London, which has added a useless annoyance and perplexity in the matter of references, by repeatedly commencing a new series, and beginning again at number one with its volumes. There is no conceivable sense in such a procedure, especially in Guy's reports, where there is no material change in the shape and size of the volumes. The Boston City Hospital has changed its style from the green cloth binding of the former volume to a plain heavy paper cover with a cloth back, in imitation of Guy's Hospital Reports.

The contents of the volume are very valuable, consisting of cases, histories and tables of a highly instructive character. Some of the cases are illustrated by series of well executed lithographs, some of which are colored. This is, in many respects, the most creditable hospital report ever issued in America, and the staff of the Boston City Hospital have reason to be proud of its contents.

E. A.

Transactions of the American Gynecological Society.— Vol. I. For the year 1876. H. D. Houghton & Co., Boston: 1877.

The value of this book consists in the articles on various gynecological subjects that it contains. The names of the writers of these articles are a guaranty that this work is of extraordinary value. Papers by Barker, Emmet, Skene, Batty, Duncan, Jenks, Parvin, Barnes (of London), Byford, Thomas, Campbell, Richardson, Chadwick, Noeggerrath, Wiltshire, Goodell, Tait, Bixby, Peaslee and Mundè form a collection of contributions seldom contained in one volume.

No detailed account of the papers submitted will be here attempted. They must be read by every physician bent on keeping pace with the development of this branch of the profession, and then he will receive instruction direct from men who have, in some instances, devoted themselves for years to the subject upon which they write.

J. H. E.

A Treatise on Hernia with a new process for its radical Cure and Original Contributions to Operative Surgery and New Surgical Instruments. *By Greenville Dowell, M. D. D. G. Brinton*, Phiadelphia, 1876.

Monographs on special subjects are common enough these days, and we suppose all of them are put forth to fill a place or supply a want in medical or surgical literature, which is noticeable to the writers thereof. If the above one is expected to fit into some vacant place, we don't know where to put it, except it be among the novelties, and lable it, not "why so," but "why made."

There may be many good and valuable suggestions in the book, but we have failed to find them. The personalities might have been omitted without detriment to the work; and in fact we think most, if not all, could have been left out without doing violence to the literature upon the subject of hernia. As to the "new operation, or the author's process" we cannot express an opinion. It seems to have been very efficient in his hands, who probably understands the workings of the process, but we have to confess to so great obtuseness of comprehension as to be unable to understand the various manipulations in the different steps of the operation, from the descriptions given, even with the aid of the very elaborate illustrative plates.

If we were to call attention to some of the very loose ways in which the subjects are treated, we would cite that of irreducible and inflamed herniæ. The causation of the latter is given as accumulation in the sac of foreign substances, such as "worms, seeds, ingesta, kicks, falls," what about these two latter getting inside of a hernial sac? The treatment would suggest itself, if not, we would refer the curious to the book. In case of irreducible hernia, we are advised to use "mercurials and alteratives to reduce the tumor," how they are to do this, is not told. We are also advised to get "complete relaxation of the neck, (neck of what is not stated) by sedatives and anodynes, as opium, chloroform, belladonna, and veratrum," how used, is not said, but we are told to use ointment of belladonna, veratrum and iodide of mercury over the tumor, to produce absorption and relaxation. How any or all of this would be expected to put an irreducible hernia into a condition to render it reducible, we cannot understand. We had supposed that a hernia which could be replaced by other means than by surgical operation should not be styled irreducible. The patient is very sagely advised to "not let himself become constipated, or the hernia impacted by cherry, water-melon, or other seeds, as they are often the cause of strangulation." The author says he thinks this variety of hernia, (the irreducible,) must be very rare, for he has never seen a case. We think he might as well have dismissed the subject at that, as to have tried to enlighten the surgical world upon a subject, of which he evidently knew so little. What has been said will apply equally well to what our author says upon incarcerated, impacted, and strangulated hernia. The causation, symptomatology, pathology and treatment all through, are something fearful if not wonderful, and we could readily believe that he has seen but little more of any kind of hernia than he confesses to in the case of the irreducible variety.

The part of the book devoted to new instruments and original contributions in operative surgery is in keeping with the other.

We were at a loss at first to know whether or not the book was intended as an advertisement for instruments and appliances, for in style and general appearance it could hardly enter the field as a rival, even of the illustrated catalogues of instrument makers and venders.

We could not commend the work to the profession, as containing information that cannot be found in better shape elsewhere, except it be to try to learn what the "author's process" is. We do not believe the work will establish the writer, with his truncated pyramid of titles, as an author, nor the publisher as a desirable book-maker.

R. G. B.

An Index of Diseases and their Treatment.—By *Thomas Hawkes Tanner, M. D., F. L. S.* Second edition. Revised by Dr. W. H. Broadbent, F. R. C. P., London. Ps. 432, 8 vo. Lindsay & Blakiston, Phila., 1877.

This volume is intended to facilitate the work of the busy practitioner. It consists of an alphabetical arrangement of all diseases, with a short description of each and of its treatment. It contains articles upon every subject, from *abscess* to *zona*. In the sections on treatment, the author gives numerical references to prescriptions in full, contained in the latter part of his book. Over 400 of these complete prescriptions are given, as a sort of appendix to the main work. In the appendix are contained some formulæ for aliments, some remarks on electro-therapeutics, a description of climates for individuals, and of mineral waters together with their analyses.

The book will prove a very useful one to the practitioner who has not the time necessary to carefully study every case for which he prescribes. The physician who studies much will not use it to any extent; but the physician who *will not* study and yet wants to know what to give in such and such cases, will probably find the work invaluable.

